# Prophylactic cranial irradiation for limited-stage small-cell lung cancer in the modern magnetic resonance imaging era may be omitted: a propensity score-matched analysis

**DOI:** 10.1093/jrr/rrae087

**Published:** 2024-10-30

**Authors:** Kei Ito, Yujiro Nakajima, Shota Minakami, Yumiko Machitori, Yukio Hosomi, Kana Hashimoto, Makoto Saito, Keiko Nemoto Murofushi

**Affiliations:** Division of Radiation Oncology, Department of Radiology, Tokyo Metropolitan Cancer and Infectious Diseases Center, Komagome Hospital, 3-18-22 Honkomagome, Bunkyo-ku, Tokyo 113-8677, Japan; Division of Radiation Oncology, Department of Radiology, Tokyo Metropolitan Cancer and Infectious Diseases Center, Komagome Hospital, 3-18-22 Honkomagome, Bunkyo-ku, Tokyo 113-8677, Japan; Department of Radiological Sciences, Komazawa University, 1-23-1 Komazawa, Setagaya-ku, Tokyo 154-8525, Japan; Division of Radiation Oncology, Department of Radiology, Tokyo Metropolitan Cancer and Infectious Diseases Center, Komagome Hospital, 3-18-22 Honkomagome, Bunkyo-ku, Tokyo 113-8677, Japan; Department of Radiology, Tokyo Metropolitan Bokutoh Hospital, 4-23-15 Kotobashi, Sumida-ku, Tokyo 130-8575, Japan; Division of Radiation Oncology, Department of Radiology, Tokyo Metropolitan Cancer and Infectious Diseases Center, Komagome Hospital, 3-18-22 Honkomagome, Bunkyo-ku, Tokyo 113-8677, Japan; Department of Radiology, Tokyo Metropolitan Bokutoh Hospital, 4-23-15 Kotobashi, Sumida-ku, Tokyo 130-8575, Japan; Department of Thoracic Oncology and Respiratory Medicine, Tokyo Metropolitan Cancer and Infectious Diseases Center, Komagome Hospital, 3-18-22 Honkomagome, Bunkyo-ku, Tokyo 113-8677, Japan; Department of Thoracic Oncology and Respiratory Medicine, Tokyo Metropolitan Cancer and Infectious Diseases Center, Komagome Hospital, 3-18-22 Honkomagome, Bunkyo-ku, Tokyo 113-8677, Japan; Division of Clinical Research Support, Tokyo Metropolitan Cancer and Infectious Diseases Center Komagome Hospital, 3-18-22 Honkomagome, Bunkyo-ku, Tokyo 113-8677, Japan; Division of Radiation Oncology, Department of Radiology, Tokyo Metropolitan Cancer and Infectious Diseases Center, Komagome Hospital, 3-18-22 Honkomagome, Bunkyo-ku, Tokyo 113-8677, Japan

**Keywords:** limited-stage small-cell lung cancer, magnetic resonance imaging, propensity score-matched analysis, prophylactic cranial irradiation

## Abstract

We aimed to clarify whether prophylactic cranial irradiation (PCI) is associated with improved outcomes in limited-stage small-cell lung cancer (LS-SCLC) in the current era of magnetic resonance imaging (MRI). Data from patients with LS-SCLC who achieved a complete response to definitive chemoradiotherapy (CRT) at two medical centers were retrospectively reviewed. Propensity score-matching was performed in a 2:1 ratio to balance the baseline characteristics of the no-PCI and PCI groups. The endpoints were the incidence of brain metastasis (BM), neurological causes of death and overall survival (OS). Overall, 80% patients underwent head MRI during the initial staging and 75 patients (no-PCI, *n* = 50; PCI, *n* = 25) were matched. Their baseline characteristics were generally well-balanced except for age; patients in the no-PCI group tended to be older. The median follow-up period was 29 months. Although the incidence of BMs tended to be higher in the no-PCI group (1-year BM occurrence: 26% vs 17%, *P* = 0.22), the incidence of multiple BMs (defined as >4 metastases) was similar between groups (1-year multiple BMs occurrence: 8% vs 9%, *P* = 0.65). The 2-year neurological causes of death and OS rate did not significantly differ between the groups (6% and 9%; *P* = 0.85; and 70% and 79%; *P* = 0.36, respectively). The 1-year occurrence of multiple BMs did not increase, even without PCI, when modern imaging modalities were integrated into the initial diagnosis, suggesting that PCI could be omitted after CRT, if MRI was incorporated into the initial diagnosis and follow-up.

## INTRODUCTION

Aggressive small-cell lung cancer (SCLC) is associated with a poor prognosis due to rapid growth and early distant and locoregional dissemination [1, 2]. Brain metastasis (BM) occurs in >10% of patients with SCLC at initial diagnosis and in >50% of 2-year survivors [3]. Prophylactic cranial irradiation (PCI) is the recommended standard treatment for limited-stage (LS)-SCLC that achieves a complete response (CR) to initial chemoradiotherapy (CRT) [4, 5]. This recommendation was based on a meta-analysis of randomized trials conducted between 1977 and 1995 that revealed a 5% overall survival (OS) advantage with PCI in trials primarily involving patients with LS-SCLC [6]. However, PCI might lead to decreased neurocognitive function [7–9].

Moreover, the randomized trials included in the meta-analysis described above had the following limitations: patients with BM were not ruled out by magnetic resonance imaging (MRI) during initial staging, 14.2% patients had extended-stage SCLC, ~25% patients included only received chemotherapy, platinum-doublet chemotherapy was not routinely administered and CR was mostly determined based on chest X-ray images [6]. Thus, the evidence of these trials differs greatly from current clinical practice. Therefore, re-evaluating the necessity of PCI has become imperative.

Based on this, we speculated that PCI could be omitted after definitive CRT, if MRI (or contrast-enhanced computed tomography [CT]) was incorporated into the initial diagnosis and follow-up of LS-SCLC. Therefore, in this study, we aimed to evaluate the necessity of PCI by comparing the 1-year rates of multiple BMs (oligo-BMs could be treated with stereotactic radiotherapy [SRT]) between patients treated with and without PCI using propensity score-matching (PSM).

## MATERIALS AND METHODS

### Patients and data acquisition

This was a retrospective review of medical databases at two institutions, which included patients with LS-SCLC who were treated with definitive CRT between January 2005 and December 2022. The inclusion criteria comprised non-metastatic SCLC diagnosed by radiographic staging, thoracic lesions treated with CRT with a curative intent, CT images of locoregional lesions with a CR to CRT, and images of the head acquired at least once after a CR was confirmed. Patients generally underwent a head MRI (or CT with contrast in some situations) to rule out BM during the initial staging according to National Comprehensive Cancer Network (NCCN) guidelines [4].

This study was approved by our Institutional Ethical Review Board (approval number 3213 in a representative institution), and informed consent was obtained in the form of an opt-out option displayed on the website.

### Treatment and follow-up

Patients were generally considered for concurrent CRT with four courses of platinum-based doublet chemotherapy with etoposide every 3 weeks and 45 Gy of thoracic radiotherapy in 30 fractions (fx) of 1.5 Gy administered twice daily for 3 weeks, with a minimum interval of 6 hours between fx. Alternatively, a dose fraction schedule of 50 Gy in 25 fx was commonly selected for patients receiving radiotherapy once-daily.

All patients, whose primary thoracic lesions responded completely to CRT, received an explanation regarding PCI. After a thorough discussion of the risks and benefits of PCI, the patients who freely opted to undergo PCI received 25 Gy in 10 fx over a period of 2 weeks. PCI was performed using lateral opposing fields and did not involve hippocampal avoidance whole-brain radiotherapy (WBRT) using intensity-modulated radiation therapy (IMRT).

Patients underwent follow-up head MRI (or CT with contrast in some situations) every 3–4 months during the first year, and every 6 months thereafter, regardless of PCI status, in accordance with NCCN guideline recommendations [4].

### Evaluation

The primary endpoint was the cumulative incidence of >4 (multiple) BMs within 1 year in both groups. The secondary endpoints were the incidences of ≤4 (oligo) BMs plus multiple BMs, oligo-BMs, neurological causes of death, progression-free survival (PFS) and OS. All endpoints of the time-to-event analyses were calculated in months from the date of definitive treatment initiation to an event or the most recent (imaging) follow-up.

### PSM analysis

Propensity scores were generated using multivariate logistic regression models that predicted treatment with or without PCI. The patients were matched based on age, Eastern Cooperative Oncology Group (ECOG) performance status (PS), clinical stage, central nervous system (CNS) staging modality, timing of concurrent chemotherapy and irradiation strategy (once vs twice daily). The absence or presence of PCI was matched in a 2:1 ratio with a caliper distance of 0.25 using the Matching package in R (R Foundation for Statistical Computing, Vienna, Austria) [10].

### Statistical analyses

The balance of baseline characteristics between the two groups was assessed using Fisher’s exact test or chi-squared test for categorical variables and Mann–Whitney U test for continuous variables. Patient death without multiple BMs was regarded as a competing risk factor. Therefore, multiple BMs were estimated using the cumulative incidence function, adjusted for the competing risk of death, and compared using the Gray test for equality. Similarly, BMs (competing risk factor: death), oligo-BMs (competing risk factors: multiple BMs and death) and neurological causes of death (competing risk factor: death due to causes of death other than neurological) were estimated using the cumulative incidence function adjusted for competing risks and compared using Gray tests. PFS and OS were estimated using the Kaplan–Meier curves and compared between the groups using log-rank tests. All data, except matching, were statistically analyzed using EZR software, v. 1.54 (Saitama Medical Center, Jichi. Medical University, Saitama, Japan) [11]. *P* < 0.05 was considered statistically significant.

## RESULTS

### Patient characteristics

During the study period, 138 patients with LS-SCLC received definitive CRT. Among them, 21 and 4 patients were excluded due to non-CR thoracic lesions on CRT, and missing imaging data of the head after confirming a CR, respectively. Ultimately, 113 patients who met the inclusion criteria were assigned to the groups based on whether they were treated without (*n* = 82) or with (*n* = 31) PCI.

Table 1 summarizes the patient characteristics. Significantly more patients aged >70 years were included in the group without PCI than in the group with PCI (51% vs 26%, *P* < 0.01). Sex, PS, CNS staging modality and chemotherapy regimens tended to differ between the groups (*P* = 0.13–0.18). BM was evaluated using MRI (plus CT) and CT only after CRT in 94 (83%) and 19 (17%) patients, respectively.

**Table 1:** Patient and tumor characteristics of all and matched patients

Characteristic	All patients			Matched patients		
	No-PCI group (*n* = 82)	PCI group (*n* = 31)	*P*-value	No-PCI group (*n* = 50)	PCI group (*n* = 25)	*P*-value
Sex Male/female	59/23	18/13	0.18	37/13	16/9	0.43
Age, y^*^ Median (range)	71 (48–86)	66 (54–75)	< 0.01	69 (48–82)	66 (55–75)	0.17
ECOG PS^*^ 0/1/2/3/4	33/42/4/3/0	19/11/0/1/0	0.18	29/19/0/2/0	15/9/0/1/0	1
T stage T1/T2/T3/T4/Tx	20/22/5/27/8	11/9/2/4/5	0.22	13/14/3/13/7	8/7/2/3/5	0.69
N stage N0/N1/N2/N3	1/20/38/23	0/6/15/10	0.87	11/31/8	4/12/9	0.16
Clinical stage^*^ I/II/IIIA/IIIB/IIIC	1/15/28/26/12	0/4/16/8/3	0.59	0/8/25/13/4	0/3/12/7/3	0.95
CNS staging^*^ MRI/CT/none	67/13/2	21/7/3	0.13	40/10/0	20/5/0	1
Value of pro-GRP Median (range)	247 (9–14 000)	470 (27–5286)	0.27	475 (9–14 000)	680 (27–5286)	0.27
CRT^*^ Concurrent Late concurrent Sequential	42 10 30	19 4 8	0.60	28 5 17	15 3 7	0.88
Chemotherapy Cisplatin/etoposide Carboplatin/etoposide Cisplatin/irinotecan	42 38 2	20 9 2	0.15	28 21 1	16 8 1	0.51
Thoracic RT^*^ Twice/once daily	56/26	23/8	0.65	35/15	18/7	1
PCI dose 25 Gy in 10 fx 30 Gy in 15 fx	0 0	28 3	NA	0 0	22 3	NA

^*^Included in propensity score model. Pro-GRP, progastrin-releasing peptide.

### Clinical outcomes of the entire cohort

**Fig. 1:**
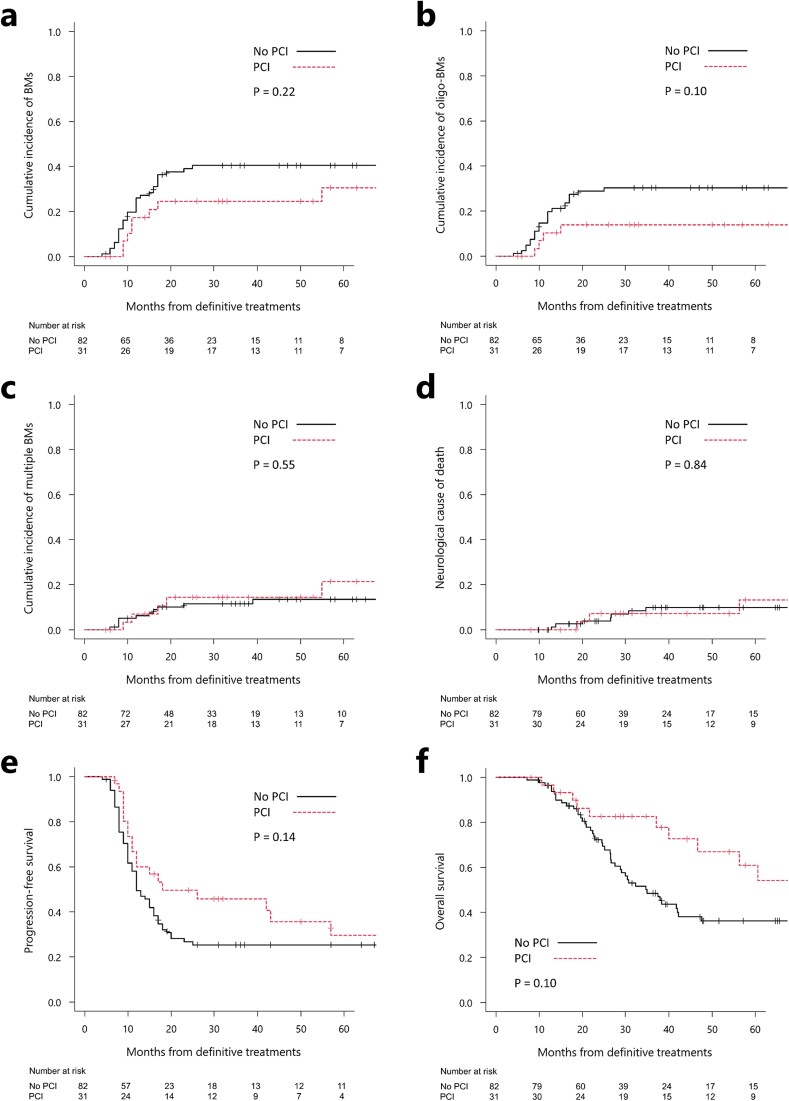
Clinical outcomes of the entire cohort with or without PCI. Cumulative incidence of BMs (a), oligo-BMs (b), multiple BMs (c) and neurological cause of death (d) after definitive treatment. Kaplan–Meier curves of progression-free (e) and overall (f) survival after definitive treatment.

The median follow-up duration after CRT was 30 months (without and with PCI: 28 [range, 7–172] and 37 [range, 8–189 months], respectively). The 1-year rates of BMs, oligo-BMs and multiple BMs without and with PCI were 26% vs 17% (*P* = 0.22), 20% vs 10% (*P* = 0.10) and 6% vs 7% (*P* = 0.55; Fig. 1a–c), respectively. The rates of neurological causes of death at 1 and 2 years were 0% and 4% and 0% and 7% (*P* = 0.84) for the groups treated without and with PCI (Fig. 1d), respectively. The 1- and 2-year, and median PFS for patients treated without and with PCI were 50% and 60%, 27% and 50%, and 12 and 18 months (*P* = 0.14; Fig. 1e), respectively. The 1- and 2-year OS rate and the median survival duration for patients treated without and with PCI were 96% and 97%, 72% and 83%, and 35 and 71 months (*P* = 0.10; Fig. 1f), respectively.

Among the 28 patients with oligo-BMs, 13, 6, 3 and 4, underwent SRT, WBRT, surgery plus adjuvant radiotherapy and chemotherapy, respectively, whereas 2 did not undergo any treatment. Among the 13 patients treated with SRT for oligo-BMs, only one patient developed multiple BMs 39 months later.

### Patient characteristics in the PSM cohort

The PSM analysis for the reduction of confounding effects identified 25 matched pairs of patients in each group (without PCI, *n* = 50; with PCI, *n* = 25). The matched groups of patients had similar baseline characteristics (Table 1), including age, PS, clinical stage, CNS staging modality, timing of concurrent chemotherapy and irradiation strategy (age, *P* = 0.17; others, *P* ≥ 0.88). BM was evaluated using MRI (plus CT) and CT only after CRT in 61 (81%) and 14 (19%) patients, respectively.

### Clinical outcomes in the PSM cohort

**Fig. 2:**
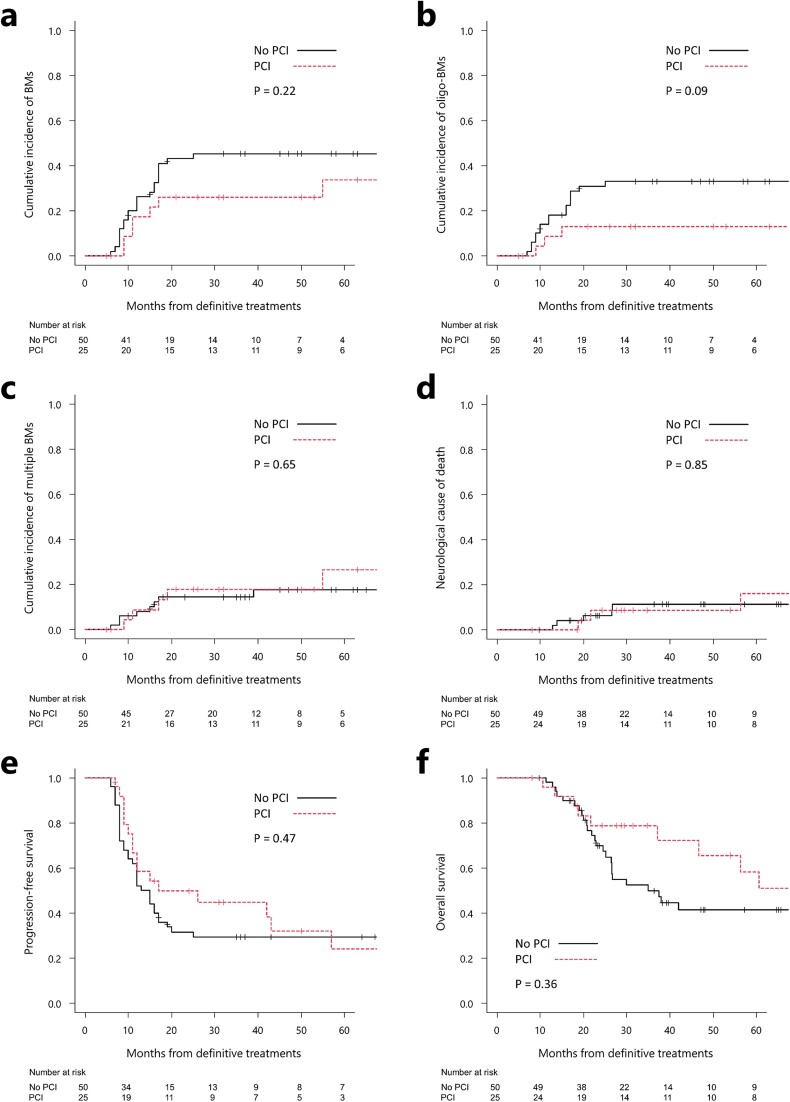
Clinical outcomes of the propensity score-matched cohort with or without PCI. Cumulative incidences of BMs (a), oligo-BMs (b), multiple BMs (c) and neurological causes of death (d) after definitive treatment. Kaplan–Meier curves of progression-free (e) and overall (f) survival after definitive treatment.

The median follow-up duration for patients without and with PCI after CRT was 29 months; (26 [7–172] vs 35 [8–159] months). The 1-year BM and oligo-BM rates in the groups without and with PCI were 26% vs 17% (*P* = 0.22) and 18% vs 9% (*P* = 0.09; Fig. 2a and b), respectively. The primary endpoint, the rates of multiple BMs at 1 year were 8% vs 9% (*P* = 0.65; Fig. 2c) in the groups without and with PCI, respectively. The rates of neurological causes of death during years 1 and 2 were identical (0%) between the groups, at 6% vs 9%, (*P* = 0.85; Fig. 2d) in the groups without and with PCI, respectively. The PFS rates at 1 and 2 years, along with the median PFS, were 52% vs 58%, 32% vs 50% and 13 vs 17 months, in the groups without and with PCI, respectively, across the groups (*P* = 0.47; Fig. 2e). The OS rates at 1 and 2 years, as well as the median survival, were 98% vs 96%, 70% vs 79% and 35 vs 71 months (*P* = 0.36; Fig. 2f) in the groups without and with PCI, respectively.

## DISCUSSION

In this study, we compared intracranial tumor control between patients treated with and without PCI using PSM analysis to determine the necessity of PCI for LS-SCLC in the current era of MRI. Our findings support the hypothesis that omitting PCI does not lead to an increase in multiple BMs within 1 year when patients are assessed using MRI at the time of initial diagnosis. Although the incidence of oligo-BMs from SCLC tended to increase when PCI was omitted, salvage SRT controlled them.

WBRT for therapeutic purposes for BMs causes cognitive dysfunction. Three phase III trials found cognitive deterioration at 2–4 months after 30 Gy of WBRT was administered in 10 or 12 fx [12–14]. However, the clinical significance of neurotoxicity after PCI at a lower dose of 25 Gy in 10 fx remains controversial. Nonetheless, a few randomized controlled trials found no significant differences in terms of neurotoxicity and quality of life between patients with and without PCI [15–17]. In contrast, a pooled analysis by the Radiation Therapy Oncology Group (RTOG) 0212 and RTOG 0214 associated PCI with a decline in self-reported cognitive functioning at 6 and 12 months after treatment with PCI compared with actual observations (odds ratios [ORs]: 3.60 and 3.44, respectively) [7]. Additionally, a recent systematic review of 3553 patients from 16 studies, including eight randomized control trials, revealed that the incidences of mild-to-moderate cognitive decline in groups with and without PCI were 8%–89% and 3%–42%, respectively [9]. Therefore theoretically, PCI may have negative effects on cognitive function, similar to those of WBRT for therapeutic purposes. As innovations in systemic therapy have prolonged the life expectancy of patients with SCLC, the need to mitigate cognitive dysfunction has become more critical than ever, regardless of whether it stems from tumor- or radiation-induced damage.

The diagnostic and treatment methods used in the landmark meta-analyses that are the basis for standard treatments [6] do not correlate with contemporary medicine. Recently published retrospective studies using PSM have reported inconsistent results [18–20]. A meta-analysis of 28 retrospective studies with 18 575 patients revealed that PCI conferred a significant benefit on OS compared to no-PCI, with an adjusted hazard ratio (HR) of 0.62 (95% CI, 0.57–0.69) [21]. Conversely, the largest PSM analysis of 648 (324 patients per group) patients with LS-SCLC reported that PCI reduced the risk of BMs (3-year cumulative rate in groups with and without PCI: 8.2% vs 38.5%; *P* < 0.01) but did not substantially prolong OS compared with active surveillance (median survival: 35.8 vs 32 months; HR, 0.90; 95% CI, 0.74–1.10) [20]. Furthermore, the present findings revealed that PCI tended to reduce BM occurrence but did not translate into significantly improved neurological causes of death and OS. When analyzing the breakdown of BMs, we found that the incidence of oligo-BMs was higher in the group without PCI, whereas that of multiple BMs was equal between the groups. Moreover, follow-up MRIs enabled the detection of a few BMs, which led to early intervention with SRT.

The present results showed that the difference in the incidence of oligo-BMs between the groups was observed ~12 months later (Fig. 2b). This was accompanied by a difference (not significant) in PFS between the groups from the same period (Fig. 2e). Similarly, a difference (not significant) in OS was observed between the groups ~22 months later (Fig. 2f). Although these group differences appear to represent a series of phenomena, the absence of differences in neurological causes of death did not provide a clear explanation for the group differences in OS. One possible explanation is that the difference in OS might be due to the fact that the group without PCI included more older patients (>70 years: 40% vs 28%, *P* = 0.17), although the patient populations were matched.

Whether SRT is appropriate for treating BMs from SCLC remains controversial. SRT has traditionally been a high-dose, extremely focal treatment delivered as a single or a few fractions, resulting in good local control and avoiding cognitive dysfunction [22, 23]. Thus, SRT is now a standard of care in managing patients with one to four BMs arising from solid tumors [22–24]. However, the rapid and numerous BMs that develop from SCLC have generally led to patients being excluded from clinical trials. Therefore, WBRT, rather than SRT, is generally recommended for BMs of SCLC [24]. In contrast, some recommendations include SRT for BMs in selected patients with SCLC [4, 25] as the outcomes of some retrospective case series have been good. A recent PSM analysis of a large-scale, international, retrospective cohort associated SRT with improved OS compared with WBRT (PSM-medians: 6.5 vs 5.2 months; *P* = 0.003); however, this should be interpreted with caution due to the retrospective nature of the data [26]. In the present study, among the 13 patients who underwent SRT for ≤4 BMs, only 1 developed multiple BMs. These findings indicate that the benefits of PCI might be attenuated in patients monitored through MRI examination and treated with SRT.

A noteworthy feature of the present study was that the primary endpoint was the occurrence of multiple BMs at 1 year. We determined that this was a suitable surrogate endpoint to evaluate the benefits of PCI by considering how long microscopic BMs would require to become apparent, if PCI was omitted. Moreover, we defined multiple BMs as events, given their indication for WBRT (as opposed to SRT). The definition of the number of multiple BMs (> 4) was determined based on the fact that several guidelines recommend SRT for ≤4 BMs [22, 24]. Thus, the present findings showed comparable rates of multiple BMs and neurological causes of death regardless of PCI status in both groups.

Nonetheless, this study had some limitations. First, the limited sample size and number of BM occurrences were unable to generate conclusive results. The lack of a significant difference in outcomes (such as OS, Fig. 2f) between the groups may have been attributed to the small sample size. Second, there may have been selection bias by the physician when explaining PCI to the patients. While we did not recommend or withhold PCI based on individual patient characteristics, a significantly higher number patients, aged >70 years, were included in the no-PCI group within the entire cohort. Third, due to the retrospective nature of the study design and data extraction procedure, the CNS imaging modality at initial diagnosis and CNS imaging follow-up method were not unified. BMs were excluded using only CT in 20% of patients at initial diagnosis. However, the results could be interpreted as reflecting real-world data, considering that prompt treatment is often necessary in daily clinical practice. Consequently, a large-scale randomized controlled trial is required to address these limitations, while the results of an ongoing phase III trial (NCT04829708) are awaited.

In conclusion, the findings of our PSM analysis suggested that the incidence of multiple BMs did not increase in the absence of PCI if MRI was incorporated into the initial diagnosis. Although omitting PCI might increase the occurrence of oligo-BM, this could be controlled by MRI during follow-up and with SRT.

## Data Availability

The datasets used and/or analyzed during this study are available from the corresponding author on reasonable request.

## PRESENTATION AT A CONFERENCE

This manuscript has not been presented elsewhere in part or in entirety.
